# Characteristics of lipids and their feeding value in swine diets

**DOI:** 10.1186/s40104-015-0028-x

**Published:** 2015-07-01

**Authors:** Brian J. Kerr, Trey A. Kellner, Gerald C. Shurson

**Affiliations:** USDA-ARS-National Laboratory for Agriculture and the Environment, Ames, IA 50011 USA; Department of Animal Science, Iowa State University, Ames, IA 50011 USA; Department of Animal Science, University of Minnesota, St. Paul, MN 55108 USA

**Keywords:** Digestion, Energy, Lipids, Peroxidation, Pigs

## Abstract

In livestock diets, energy is one of the most expensive nutritional components of feed formulation. Because lipids are a concentrated energy source, inclusion of lipids are known to affect growth rate and feed efficiency, but are also known to affect diet palatability, feed dustiness, and pellet quality. In reviewing the literature, the majority of research studies conducted on the subject of lipids have focused mainly on the effects of feeding presumably high quality lipids on growth performance, digestion, and metabolism in young animals. There is, however, the wide array of composition and quality differences among lipid sources available to the animal industry making it essential to understand differences in lipid composition and quality factors affecting their digestion and metabolism more fully. In addition there is often confusion in lipid nomenclature, measuring lipid content and composition, and evaluating quality factors necessary to understand the true feeding value to animals. Lastly, advances in understanding lipid digestion, post-absorption metabolism, and physiological processes (e.g., cell division and differentiation, immune function and inflammation); and in metabolic oxidative stress in the animal and lipid peroxidation, necessitates a more compressive assessment of factors affecting the value of lipid supplementation to livestock diets. The following review provides insight into lipid classification, digestion and absorption, lipid peroxidation indices, lipid quality and nutritional value, and antioxidants in growing pigs.

## World production of lipid sources

Global production of vegetable oils has increased dramatically over the last 20 years with approximately 168 million metric tonnes produced in 2014. The primary vegetable oils produced in the world include palm oil (35 % of the total production), soybean oil (26 %), rapeseed/canola oil (15 %), and sunflower oil (9 %). Other vegetable oils account for only about 15 % of the market, with palm kernel oil, cottonseed oil, peanut oil, coconut oil, olive oil, and corn oil rounding out the 10 vegetable oils produced in the greatest quantities worldwide [1]. Production of animal fats has also increased, although less in magnitude than for vegetable oils. Fats obtained from the rendering industry represent inedible lipids that are recycled into animal feeds as highly concentrated energy sources. The National Renderers Association [2] reported that the U.S. rendering industry produces about 5 million metric tonnes of edible and inedible tallow (57 % of U.S. rendered fats), yellow grease (19 %), lard and choice white grease (12 %), and poultry fat (10 %). In addition to these primary lipid sources, the U.S. biodiesel industry produces by-products including crude glycerin, fatty acid distillate, glycerin bottoms, and oleo-lipids. The oilseed industry produces products such as lecithin, soapstock, acid oil, and fatty acid distillate, all of which find their way directly into livestock and poultry feeds or indirectly through further processing or blending with other lipids. Lastly, lipids produced by the food industry include dried fats, mono-and diglycerides, and emulsifiers that may be available to the feed industry for use as potential energy sources.

## Lipid classification

Lipids are a group of structurally diverse, water-insoluble, organic-solvent-soluble compounds. Lipids have hydrocarbon chains or rings as a major part of their chemical structure, with the primary types of hydrocarbons being fatty acids (**FA**) and steroids. Fatty acids are linear, aliphatic monocarboxylic acids [R-(CH_2_)_n_COO-], and almost always have an even number of carbons. Unsaturated FA may contain one or more cis double bonds. No conjugated double bond lipids are found in nature except for conjugated linoleic acid. Furthermore, there are very few naturally produced ‘trans’ fats, but some ‘trans’ fats can be produced as a result of hydrogenation processes which occur in the rumen and during industrial processing.

A number of conventions exist for naming individual FA, including trivial names, systematic names, as well as describing them by the number of carbons in the FA chain followed by the number of double bonds [3–5]. The arrangement of double bonds within a FA is also subject to two different classification systems. The International Union of Pure and Applied Chemistry system classifies lipids based on the position of the double bond relative to the carboxyl carbon (e.g. linoleic acid is Δ9,12-18:2 or cys, cys-9,12-18:2). Another classification system is based on the position of the double bonds relative to the methyl terminal of the FA, using either the ω (omega) or the n- (“n-minus”) naming system, where ω or n- counts the number of carbon atoms from the methyl carbon as position-1. Thus with this system, linoleic acid is defined as 18:2 ω6 or 18:2 n-6. Within the ω or n- system, there are three main families of naturally occurring FA based on the position of the first double bond. The most common series is ω3, ω6, and ω9 (n-3, n-6, and n-9, respectively). The three ω3 FA that are of keen nutritional interest are α-linolenic acid (18:3), eicosapentaenoic acid (20:5 or **EPA**), and docosahexaenoic acid (22:6 or **DHA**). These three ω3 FA are essential for normal growth and health, and have been associated with cardiovascular health, reduced inflammation, and normal development of the brain, eyes, and nerves [6–8]. The two ω6 FA that are of utmost nutritional interest are linoleic acid (18:2) and arachidonic acid (20:4), which are converted to ω-6 eicosanoids [9]. The two ω9 FA that receive most attention are oleic acid (18:1) and erucic acid (22:1). Oleic acid is found in high concentrations in olive oil and many other monounsaturated lipids, while erucic acid has been associated with heart lesions in rats and reduced weight gain in farm animals [10]. Unlike the ω3 and ω6 FA, the ω9 FA are not classified as essential FA because they can be created from unsaturated FA, and because they lack the ω6 double bond, they are not important in the formation of eicosanoids. Although it has been difficult to produce overt signs of an essential FA deficiency in pigs [11], there is renewed interest in the level and ratio of these FA in both human and animal nutrition [12, 13]. A general description and source of common FA is shown Table 1.

**Table 1:** Descriptions of common fatty acids

Common name	Carbons	Double-bonds	Scientific name	Common source
Formic	1	0	methanoic acid	insect stings
Acetic	2	0	ethanoic acid	vinegar
Propionic	3	0	propanoic acid	bacteria fermentation
Butyric	4	0	butanoic acid	butter fat
Caproic	6	0	hexanoic acid	goat fat
Caprylic	8	0	octanoic acid	coconut oil
Capric	10	0	decanoic acid	coconut oil
Lauric	12	0	dodecanoic acid	coconut oil
Myristic	14	0	tetradecanoic acid	palm kernel oil
Palmitic	16	0	hexadecanoic acid	palm oil
Palmitoleic	16	1	9-hexadecenoic acid	animal fats
Stearic	18	0	octadecanoic acid	animal fats
Oleic	18	1	9-octadecenoic acid	olive oil
Ricinoleic	18	1	12-hydroxy-9-octadecenoic acid	castor oil
Vaccenic	18	1	11-octadecenoic acid	butterfat
Linoleic	18	2	9,12-octadecadienoic acid	grape seed oil
α-Linolenic	18	3	9,12,15-octadecatrienoic acid	flaxseed (linseed) oil
γ-Linolenic	18	3	6,9,12-octadecatrienoic acid	borage oil
Arachidic	20	0	eicosanoic acid	peanut oil, fish oil
Gadoleic	20	1	9-eicosenoic acid	fish oil
Arachidonic	20	4	5,8,11,14-eicosatetraenoic acid	liver fats
Eicosapentaenoic	20	5	5,8,11,14,17-eicosapentaenoic acid	fish oil
Behenic	22	0	docosanoic acid	rapeseed oil
Erucic	22	1	13-docosenoic acid	rapeseed oil
Docosahexaenoic	22	6	4,7,10,13,16,19-docosahexaenoic acid	fish oil
Lignoceric	24	0	tetracosanoic acid	some in most fats

Sources: [5,188]

As a subgroup of lipids, the terms fat and oil are often incorrectly used interchangeably. Technically, oil is the term generally used to refer to lipids that are liquid at room temperature and of vegetable origin, while fat refers to lipids that are generally solid at room temperature and of animal origin. For example, flaxseed, soybean, and sunflower oils have a melting point between -17 to -24°, while corn, canola, and olive oils have a melting point between -5 to -10 °C. In contrast, poultry fat has a melting point of approximately 25 °C, while lard and tallow have a melting point between 35 to 45 °C. Differentiation of lipids by melting points is not always consistent, however, where coconut and palm oils are named solely on their vegetable origin rather than their physical properties because these oils have melting points between 25 to 35 °C.

Most lipids are primarily composed of triglycerides, but they may also contain other lipid compounds which may affect their chemical and physical properties, as well as their energy value to animals. Sterols have high melting points, are colorless and somewhat inert, and represent a minor proportion in natural lipids. Most of the unsaponifiable material present in lipids consists of sterols, with cholesterol being the main sterol component in animal fats and fish oil. Sterols are also found in vegetable oils, but only in trace amounts. Waxes are high-melting point esters of fatty alcohols and fatty acids that commonly have a chain length of 8 carbons or longer, and have low solubility in oils. Waxes tend to solidify after a period of time, giving oil a cloudy appearance, unsightly threads, or a layer of solidified material. Phospholipids (referred to as phosphatides by oil processors) consist of polyhydric alcohols esterified with fatty acids and phosphoric acid, which are further combined with nitrogen-containing compounds. Two phospholipids commonly found in vegetable oils are lecithins and cephalins. Tocols are also found in plant-based lipids, with tocopherols and tocotrienols considered to be natural antioxidants. Tocopherols have a saturated side chain whereas tocotrienols have an unsaturated side chain, and as a result, tocopherols have more vitamin E or effective antioxidant activity than tocotrienols. Phospholipids combined with a small quantity of carbohydrates and resins, are commonly called gums.

Analysis of the lipid content in a feedstuff, diet, digesta, or fecal matter is determined by multiple methods. Lipid analysis methods vary in solvent type (ether, hexane, or chloroform), extraction time, temperature, pressures, and sample dryness. Crude fat extraction methods typically do not completely extract FA, especially if they are linked to carbohydrates or proteins, or are present as salts of divalent cations [14]. Extraction of lipids by acid-hydrolysis is believed to correct for this deficiency by breaking FA away from tri-, di-, and mono- acylglycerides, lipid-carbohydrate bonds, lipid-protein bonds, sterols, and phospholipids, resulting in a more complete extraction. Therefore, the concentration of lipids in feedstuffs, diets, digesta, or feces is usually higher by using acid-hydrolysis than by crude fat extraction methods [11, 14, 15], although this is not always the case [16]. Fat extraction method and solvent used may also have an effect on the digestibility coefficient of lipids in a diet or feedstuff [17]. Selection of the appropriate laboratory method is essential for accurate determination of lipid composition as well as to ensure that a lipid product meets trade specifications and the requirements of a buyer. Table 2 describes some of the most common lipid composition measures used in animal nutrition research, but there are no standards or consistency on which measures are reported in the scientific literature. Likewise, these indices are generally used to ensure that the lipid products meet trading specifications, but provide little or no information on the extent of lipid peroxidation and relative feeding value [18].

**Table 2:** Lipid quality indices

Item	Description
Color	Quantified relative to the Fat Analysis Committee (FAC) standard, ranging from 1 (light) to 45 (dark).
Fatty acid profile	Relative amounts of individual fatty acids in a sample.
Free fatty acids	Amount of fatty acids not bound to the glycerol backbone in a triglyceride.
Insolubles	Amount of sediment in a sample. For example, fiber, hair, hide, bone, or soil.
Iodine value	Measure of chemical unsaturation, expressed as grams of iodine absorbed by 100 g of fat. The iodine value can be calculated based upon fatty acid profile.
Moisture	Amount of moisture in a sample.
Nonelutable material	Reflects the total amount of non-nutritional material; includes moisture, impurities, unsaponifiable material, glycerol, and oxidized and polymerized fats.
Saponification value	An estimate of the average molecular weight of the constituent fatty acids in a sample, defined as milligrams of KOH required to saponify 1 g of lipid. The greater the saponification value, the lower the average chain length.
Titer	The solidification point of fatty acids in lipids, which is an important characteristic in producing soaps or fatty acids.
Total fatty acids	The total of both free fatty acids and fatty acids combined with glycerol.
Unsaponifiables	A measures of material in the lipid that will not saponify (form a soap) when mixed with caustic soda (NaOH or KOH). Examples include: sterols, hydrocarbons, pigments, fatty alcohols, and vitamins.

## Overview of lipid digestion and absorption

Digestion of dietary lipids begins with salivation, mastication, and a release of lingual lipase in the mouth [19]. Upon release from the serous glands of the tongue, lingual lipase hydrolyzes a free FA from the triacylglycerol structure at the sn-3 position as the digesta travels to stomach [20], where ‘sn’ refers to the stereochemical numbering of the glycerol backbone. Once the digesta reaches the stomach, gastric lipase continues the hydrolysis of dietary lipids by releasing mainly short chain FA that were esterified as part of the triacylglyceride [20]. Despite hydrolysis by these two lipases, the lipids entering the upper duodenum are still greater than 70 % triacylglycerides [19]. Therefore, the small intestine is the location where the majority of dietary lipid digestion occurs [21].

Digestion of lipids in the small intestine involves two key constituents: bile salts and pancreatic lipase. Bile salts are formed from cholesterol in the liver and are subsequently concentrated and stored in the gallbladder [22]. The release of bile salts into lumen takes place when and where water/oil emulsion occurs, and is caused when circulating levels of cholecystokinin, a peptide hormone, is increased [22]. While bile salts are essential for micelle formation, when they are released into the intestinal lumen they initially cause inhibition of pancreatic lipase activity. This inhibition is due to bile salts physically blocking pancreatic lipase from coming in contact with lipid droplets in the lumen [19]. Colipase reverses the inhibition of bile salts by binding to pancreatic lipase, which once adjoined, can adhere to the surface of the lipid droplet [19]. Once pancreatic lipase is adhered to the lipid droplet by the binding of colipase, it enzymatically cleaves the ester bond of the triacylglycerol at the sn-1 and sn-3 positions [23]. The resulting enzymatic hydrolysis creates two free FA and a monoacylglycerol with a FA esterified at the sn-2 position. This enzymatic activity occurs very quickly, and produces free FA and monoacylglycerols at a faster rate than subsequent micelle incorporation [24]. Phospholipids, which are resistant to hydrolysis via pancreatic lipase, undergo digestion via phospholipase A_2_ [25]. Phospholipase A_2_ enzymatically releases the FA from the sn-2 position yielding lysophosphoglycerides and free FA [25]. Colipase shuttles the recently hydrolyzed products from the lipid droplets in the lumen to micelles being formed that contain bile salts [19].

**Fig. 1:**
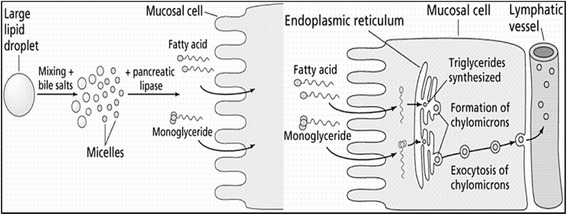
General schematic of lipid digestion and absorption

Once this enzymatic activity occurs, a complex of water soluble lipid material forms a micelle [26]. Micellar formation occurs from the actions of bile salts and phospholipids which are secreted in bile from the gallbladder. Bile salts have a polar end which faces toward the water milieu of the digesta and lumen, and a nonpolar end which face the center of the micelle. The orientation of bile salts along with phospholipids creates a hydrophobic center and hydrophilic edges for the micelle conglomeration [19]. When incorporating lipid material into the structure, some evidence supports that micelles have a higher affinity for polyunsaturated FA (**PUFA**) and saturated monoacylglyerols [27, 28]. Once the mixed micelle is formed, it transverses across the lumen to the unstirred water layer next to the apical membrane of the enterocyte [19]. The formation of a micelle solves the problem of dietary derived lipids being hydrophobic in the aqueous environment of the intestinal lumen [26]. This allows for the lipid material now contained in a mixed micelle to easily pass across the unstirred water layer, as well as increase the concentration of free fatty acids, monoacylglycerols, and other lipid materials near the absorptive surface of the enterocyte by 100 to 1,000 times [29]. A simplistic overview of lipid digestion and absorption is depicted in Fig. 1.

Due to a gradient that has been created by concentrating lipid material in micelles, lipid constituents can passively diffuse by a non-energy dependent process into the enterocyte [30]. There is also evidence to support a carrier dependent process of absorption across the lipid bilayer of the enterocyte when concentration of lipid content in the lumen is low [31]. This dual mechanism for lipid absorption has been theoretically proposed to maintain required levels of essential FA when dietary lipid intake is low, but it is unknown if carrier mediated transportation is important when dietary lipid intake is normal or high [32]. Micelles maintain an equilibrium relationship with other micelles due to the churning action and structure of the intestine, which causes almost continous contact among the epithelium, micelles, and lipid droplets [19]. This high degree of contact partitions lipid constituents from more highly populated to less populated micelles [19]. This partitioning causes micelles to evenly acquire and distribute lipid constituents, which ultimately means that the limiting factor of lipid digestion in the lumen of the small intestine is micelle saturation [19]. Shuttling of lipid constituents from the micelles across the unstirred water layer is a chain reaction that depends on low cellular concentration of lipids at the enterocyte [32]. Intestinal FA binding proteins increase the uptake of FA by binding to free FA and then entrapping FA in the vicinity of the apical membrane [33]. Bile salts are efficiently recycled via absorption in the lower ileum and transported back to the liver for re-use in subsequent lipid digestion [34].

Once diffusion into the enterocyte has occurred, FA are re-esterified in the endoplasmic reticulum by the glycerol-3-phosphate pathway or the monoacylglycerol pathway [35]. After re-esterification into a triacylglyceride, multiple triglycerides and cholesterol esters are packaged into a chylomicron [36]. Chylomicrons contain 80 to 95 % triacyglcerides, 2 to 7 % cholesterol, and 3 to 9 % phospholipids [19]. The exterior of the chylomicron has a phospholipid bi-layer and apolipoproteins which increase solubility and enzymatic recognition [26]. Chylomicrons then enter the blood circulatory system via the lymphatic system at the thoracic duct [26].

Once chylomicrons enter the blood stream, they can be stored in adipocytes, or oxidized by myofibers and other cells [19]. If insulin and other anabolic hormones are elevated, chylomicrons will be directed to adipocytes for storage [37]. This process is regulated by the stimulation effect of insulin on adipocyte lipoprotein lipase, while the isoform of lipoprotein lipase in muscle cells is not stimulated by insulin [37]. Therefore, the multi-functional enzyme lipoprotein lipase will be expressed in the capillary lumen of adipocytes to process triglyceride-rich chylomicrons and other lipoproteins [37]. Fatty acids are passively diffused individually, and then re-esterified for storage as a triacylglyceride in adipocytes [19].

In contrast to long-chain triacylglycerols which contain FA with 16 to 20 carbons, medium-chain triacylglycerols predominantly contain saturated FA with 8 and 10 carbons. Once these FA are rapidly cleaved by lipases, they have high water solubility and are readily absorbed into mucosal cells, even in the presence of low amounts of intraluminal bile salts and pancreatic lipases for chylomicron formation. These medium-chain FA are then bound to albumin and transported by the portal venous system to the liver, with a carnitine-independent transport into mitochondria for subsequent oxidation. [38–40].

## Lipids in swine diets

Supplemental fats and oils are commonly added to swine diets to increase energy density of the diet, but may also reduce dust, supply fat soluble vitamins and essential FA, and improve diet palatability [41, 42]. Composition of lipids utilized in swine diets is highly variable. Not only are there ‘new’ lipids becoming available (e.g. distiller’s corn oil), but there are also by-products from the vegetable oil processing and the biodiesel industry that can be blended with commonly used fats and oils resulting in a plethora of animal-vegetable blends. Approximate FA composition of several common, unblended, lipid sources used in swine diets is shown in Table 3.

**Table 3:** Approximate fatty acid composition of various fats and oils

	Fatty acid																		
Source	6:0	8:0	10:0	12:0	14:0	16:0	18:0	20:0	22:0	16:1	18:1	18:2	18:3	20:1	22:1	20:4	20:5	22:5	22:6
Algae	-	-	-	-	7	18	2		-	6	9	8	8	-	-	15	9	-	15
Canola	-	-	-	-	-	4.0	1.8		-	0.2	56.1	20.3	9.3	1.7	0.6	-	-	-	-
Coconut	0.5	7.8	6.7	43.8	16.8	8.4	2.5	0.1	0.3	-	5.9	1.7	-	-	-	-	-	-	-
Corn	-	-	-	0.2	0.2	10.6	1.9	0.4	0.1	0.1	27.3	53.5	1.2	0.1	-	-	-	-	-
Flaxseed	-	-	-	-	-	5.3	4.1	-	-	-	20.2	12.7	53.3	-	-	-	-	-	-
Lard	-	-	0.1	0.2	1.3	23.8	13.5	0.2	-	2.7	41.2	10.2	1.9	1.0	-	-	-	-	-
Menhaden	-	-	-	1.0	10.0	18.0	5.0	-	-	10.5	14.5	2.2	1.5	1.3	0.4	5.0	13.2	4.9	10.0
Olive	-	-	-	-	-	11.3	2.0	0.4	-	1.3	71.3	9.8	0.8	0.3	-	-	-	-	-
Palm	-	-	-	-	1.1	44.0	4.5	0.4	-	0.1	39.2	10.1	0.4	-	-	-	-	-	-
Poultry	-	-	-	0.1	0.9	21.6	6.0	-	-	5.7	37.4	19.5	1.0	1.1	-	0.1	-	-	-
Soybean	-	-	-	-	0.1	10.3	3.8	0.3	0.3	0.2	22.8	51.0	6.8	0.2	-	-	-	-	-
Sunflower	-	-	-	-	-	5.4	3.5	0.4	0.7	0.2	45.3	39.8	0.2	-	-	-	-	-	-
Tallow	-	-	0.1	0.9	3.7	24.9	18.9	0.2	-	4.2	36	3.1	0.6	0.3	-	-	-	-	-

Sources: [5,11,189,190]

**Fig. 2:**
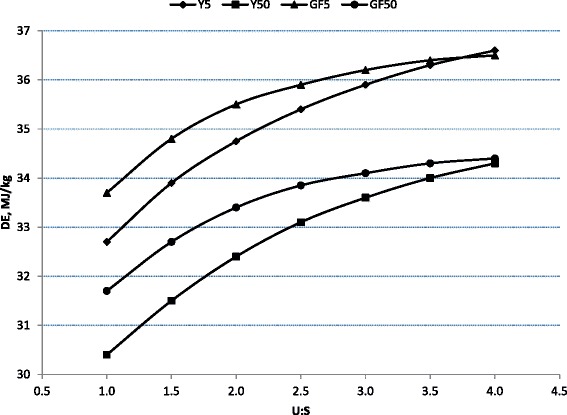
Impact of unsaturation:saturation (U:S) index and percentage free fatty acids (5 versus 50 %) on digestible energy (DE) in young (Y) or growing-finishing (GF) pigs [58]

Fats and oils are considered to be highly digestible energy sources for pigs [43–50]. However, their source and dietary inclusion rate may affect nitrogen digestibility and retention, and amino acid absorption [45, 46, 48, 51–54]. In general, the apparent total tract digestibility of lipids in nursery pigs increases with age [55, 56] with digestibility of animal fats (lard and tallow) increasing to a greater extent with age compared with vegetable oils [44–47]. In addition to animal age, the other main factors affecting the digestibility of lipids, and its subsequent energy value to pigs, is carbon chain length, degree of saturation, and free fatty acid (**FFA**) content, especially in young pigs, Fig. 2 [57, 58]. These responses are supported by others [54, 59–61] who reported that digestibility of FFA is lower than that of triglycerides, which coincides with a lower digestible energy content of lipids with increasing concentrations of FFA [57, 62, 63]. In contrast, DeRouchey et al. [64] reported that FA digestibility was not affected by FFA concentrations in choice white grease fed to nursery pigs. Recently, we reported that nursery pigs fed a diet containing 10 % of a 95 % FFA product derived from either soybean oil or corn oil had little effect on lipid digestibility and subsequent digestible or metabolizable energy (**DE** and **ME**, respectively) content in young pigs, while increasing concentrations of FFA in distiller’s corn oil reduced DE, and DE as a percentage of gross energy (**GE**), even though lipid digestibility appeared to be unaffected [65].

Factors associated with the origin and processing of lipid products (i.e. human food or agricultural industries) may also affect lipid digestibility and utilization. These factors include the concentration and FA composition of mono- and di-glycerides, acid oils, soap stocks, presence of emulsifying agents, and degree of hydrogenation. Tullis and Whittemore [66] suggested that the poor digestibility of hydrogenated tallow in swine diets is likely due to the high concentration of stearic acid. More recently, Gatlin et al. [67] reported that apparent fat digestibility decreased linearly as the dietary amount of fully hydrogenated tallow or choice white grease fat increased, suggesting that the digestibility of fully hydrogenated animal fats is approximately zero. Lecithin has been shown to have little impact on lipid and energy digestibility or growth performance in swine [68–72]. Kerr and Shurson [65] reported that lecithin had no effect on ether extract (**EE**) digestibility when added to soybean oil or soybean oil-FFA, but it interacted with FFA level, reducing DE content and DE as a percentage of GE and ME content when added to soybean oil-FFA, but not when added to soybean oil. Lysolecithin (hydrolyzed lecithin in which the sn-2 FA is removed) has been shown to improve digestibility of soybean oil, lard, tallow and coconut oil, but had minimal effects on pig growth performance [49]. During a 28 d trial, Xing et al. [73] reported an increase in digestibility of lard fed to nursery pigs supplemented with 0.05 % lysolecithin on d-10, but no effect on energy digestibility. On d-28, however, neither lipid nor energy digestibility was affected by lysolecithin supplementation, but there appeared to be a slight improvement in piglet weight gain [73]. Averette-Gatlin et al. [67] reported no effect of lysolecithin on digestibility of partially hydrogenated choice white grease fed to finishing pigs.

Lipid digestibility also relates to the positioning of the FA on the triglyceride molecule [74, 75]. However, determining the FA positioning on the glycerol molecule is difficult [76], and as a consequence, information on the effect of specific FA on the sn-1, sn-2, or sn-3 position of glycerol regarding lipid digestibility is sparse. In general, it is believed that long-chain FA on the sn-1 and sn-3 positions are absorbed less efficiently than long-chain FA bound on the sn-2 position, due to their hydrophobic characteristics. This relationship is supported by Bracco [28] who suggested that the presence of a long-chain saturated FA (**SFA**) at the sn-1 and sn-2 positions of a triglyceride is partially responsible for the poor absorption of cocoa butter. Furthermore, Smink et al. [77] reported that randomization of palmitic acid to the sn-2 position in palm oil had a positive effect on its digestibility in broilers. In swine, the effect of FA position is less clear. Scheeder et al. [78] reported that FA position of either low- or high-PUFA lipids had no impact on FA composition of depot fat in growing pigs, which suggests no impact on lipid digestibility. These results were supported by Innis et al. [79] who reported that the FA composition of adipose tissue was only slightly influenced by the triglyceride structure of various lipids. In contrast, Innis and Dyer [80] reported that the FA on the sn-2 position is conserved during digestion and absorption, and subsequently, it is reassembled to chylomicron triglycerides. Fatty acid location on the glycerol molecule may also be important because long-chain non-esterified FA at the sn-1 and sn-3 positions may have reduced absorption due to their tendency to form insoluble soaps with divalent cations [81, 82].

The NRC [11] estimates of DE content of various fat and oil sources based on the classic research by Wiseman et al. [83] and Powles et al. [57, 63, 84], where DE kcal/kg = [(36.898 – (0.005 × FFA, g/kg) – (7.330 × e^-0.906×U:S^))/4.184], and ME is subsequently calculated as 98 % of DE. Even though research studies [54, 85–87] have shown that the DE and ME content of various refined lipids in swine are similar to values reported in the NRC [88], the effect of fatty acid carbon chain length of less than 16 or greater than 18 (as utilized by [57, 63, 83, 84]), the specific location of the unsaturated or saturated fatty acids on the glycerol backbone [77], the effect of quality (moisture, insoluble, and unsaponifiables-**MIU**, nonelutable material-**NEM)**, and the extent of peroxidation on energy value among lipid sources has not been well established. Beyond nursery pigs [44–47, 55, 56], there is little comparative data available to compare lipid digestibility or energy values of lipids between nursery, growing, finishing, and mature (gestating or lactating sows), similar that which has been conducted for amino acids or fiber [89,90]. However, it is worthy to note that the NE of soybean oil or choice white grease was not found to be different between growing and finishing pigs [91] suggesting that digested lipids may be used at a relatively constant rate for incorporation into body lipids or for ATP synthesis.

The net energy (**NE**) content of dietary lipids also needs to be more accurately determined. In the NRC [11], NE was calculated as 88 % of ME based upon research by van Milgen et al. [92]. This approach was based on the NE of dietary lipid sources ranging from 6.18 to 7.56 Mcal/kg, with higher values assigned to lipids with greater unsaturated to saturated fatty acid ratios [11]. It is generally assumed that the efficiency of converting ME to NE for lipids is high [93–95]. This assumption is supported by Sauvant et al. [96] who reported that soybean oil and choice white grease have an NE content of 7.12 Mcal/kg, and [92] who reported that vegetable oil has an NE content of 7.02 Mcal/kg. However, major discrepancies in the NE content of dietary lipids have been reported. Kil et al. [91] reported that the NE content of soybean oil was 4.68 Mcal/kg and choice white grease was 5.90 Mcal/kg, while Galloway and Ewan [97] reported that the NE content of tallow was 4.18 Mcal/kg. It is interesting to note that in NRC [88], generalized equations based on constituents of the ingredient including ME, ash, and acid detergent fiber [98, 99] were used for calculating NE content. As a result, NE values for dietary lipid sources ranged from 4.93 Mcal/kg to 5.37 Mcal/kg, with higher values assigned to lipids having a greater unsaturated to saturated fatty acid ratio [88]. In addition, the post-absorptive utilization efficiency of FA is determined whether it is used for a product (body lipid deposition) or a process (ATP production). The efficiency of absorbed dietary lipids is much higher if deposited as body lipids (approximately 90 %) versus oxidized for maintenance (approximately 62 %; [92]).

## Lipid peroxidation

**Fig. 3:**
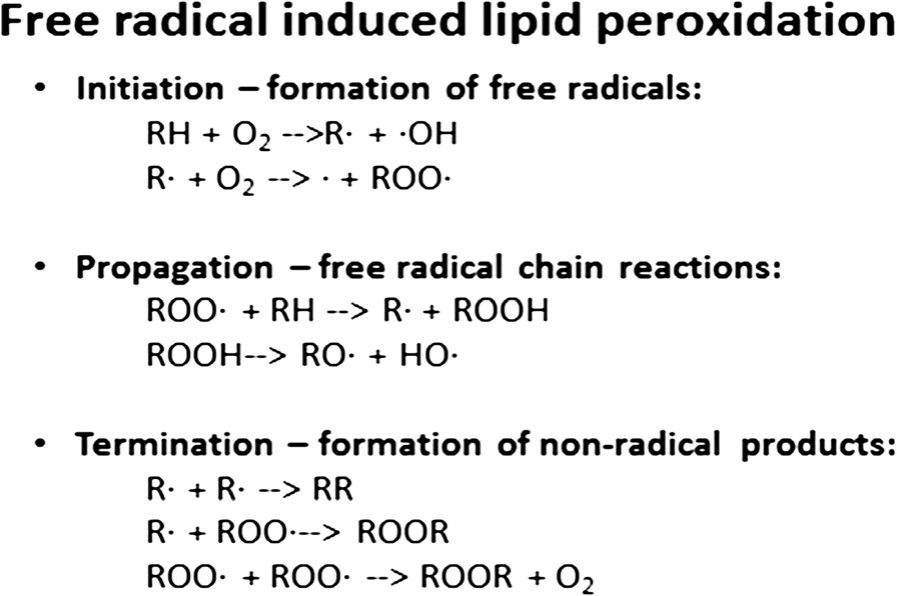
Generalized lipid peroxidation process. [“H” = α-methylenic hydrogen atom; “R” = alkyl group of an unsaturated lipid molecule; “RH” = lipid molecule; “O_2_” = oxygen (initiator); “R•” = alkyl radical; “RO•” = alkoxyl radical; “ROO•” = peroxy radical; [105]]

**Fig. 4:**
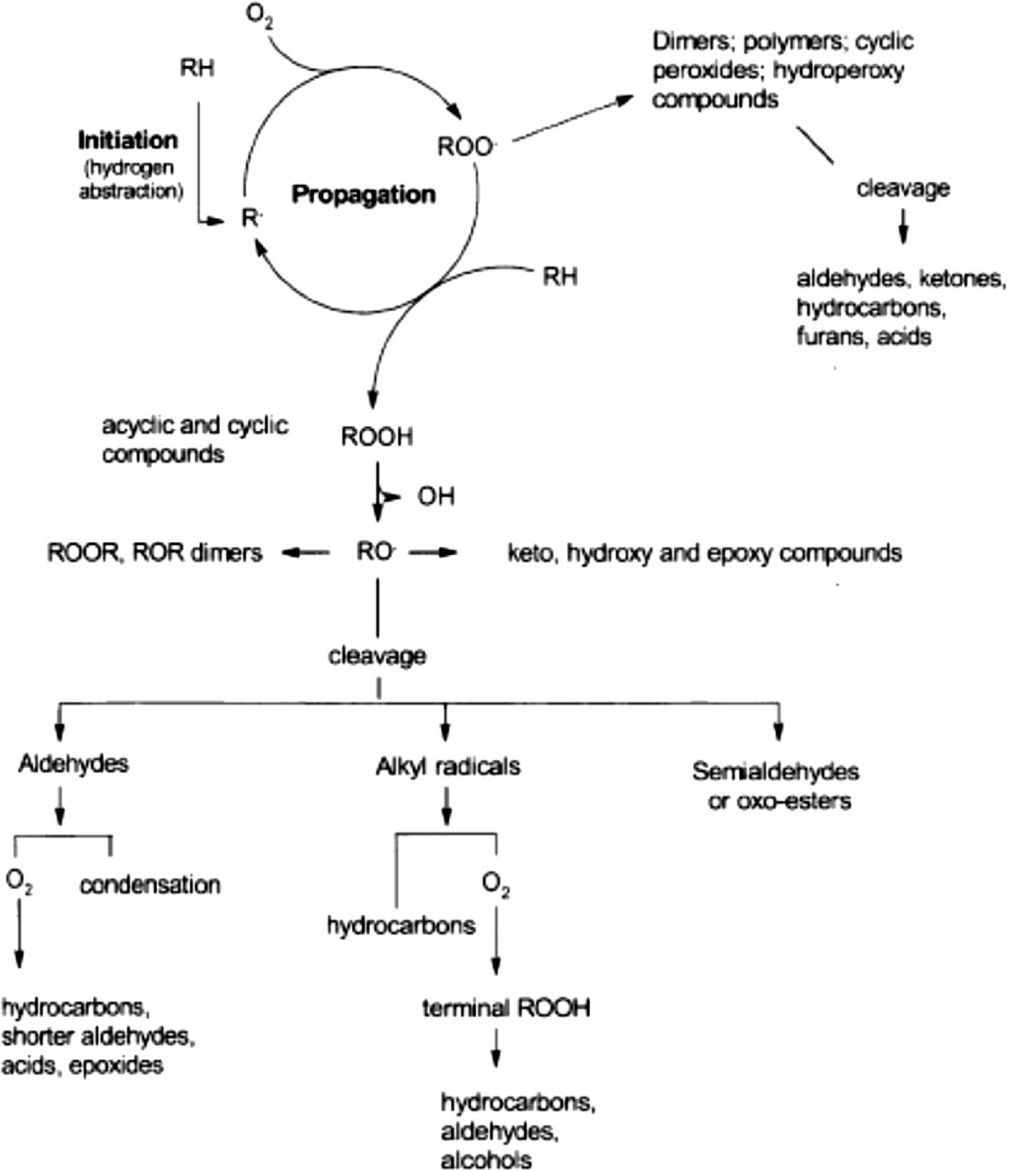
Generalized lipid peroxidation process [106]

**Fig. 5:**
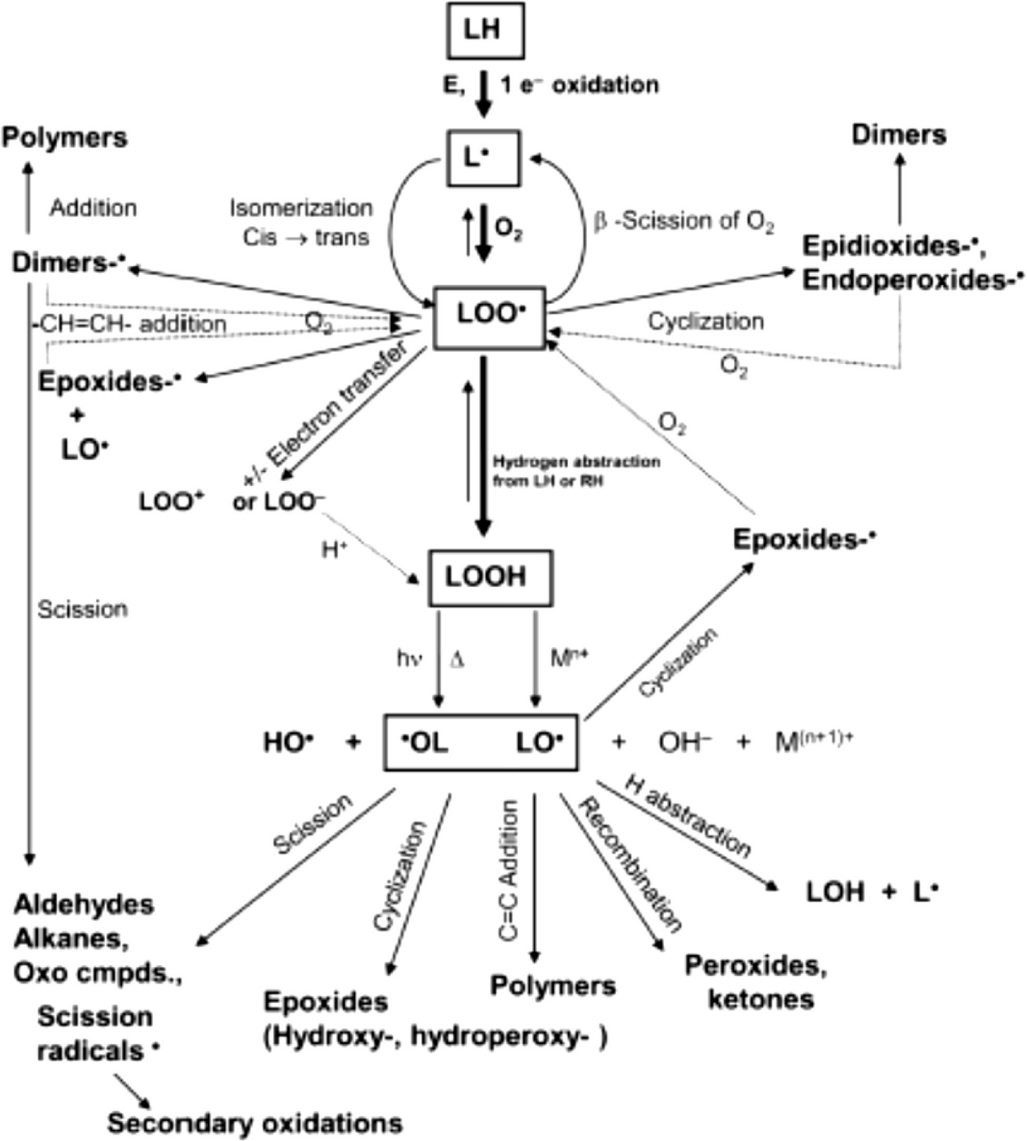
Integrated scheme for lipid oxidation [107]

In their unaltered state, lipids are primarily comprised of saturated or unsaturated FA linked to a glycerol backbone. However, factors such as the degree of saturation, temperature, as well as exposure to oxygen, transition metals, undissociated salts, water, and other non-lipid compounds can affect the ultimate composition of a lipid over time [100–102]. Lipid peroxidation is a complex and dynamic process that degrades and produces numerous peroxidation compounds over time [103]. The lipid peroxidation process has been classically described in three phases: (1) the initiation phase involves the formation of free lipid radicals and hydroperoxides as primary reaction products, (2) the propagation phase where the hydroperoxides formed are decomposed into secondary peroxidation products, and (3) the termination phase which involves the formation of tertiary peroxidation products ([101, 104–106]; Figs. 3 and 4**)**. With advances in understanding and measuring oxidation reactions with more sophisticated chromatography and spectroscopy methods, a more integrated paradigm has emerged to recognize the complexity of lipid oxidation (Fig. 5; [102, 107]).

Peroxidation of lipids is caused primarily by the attack of an oxygen molecule on unsaturated fatty acids. The rate of oxygen uptake by a fatty acid increases with the degree of unsaturation, but the mechanisms of peroxidation for the various types of FA are different [108]. Although saturated and monounsaturated FA (**MUFA**) are essentially resistant to peroxidation, saturated FA can undergo peroxidation, but at a much slower rate. At temperatures above 100 °C, however, oxygen can attack the β-carbon of SFA and MUFA, to produce hydroperoxides as the primary peroxidation product. Similar to that for PUFA, SFA and MUFA have increased susceptibility to peroxidation with increasing carbon chain length [109]. In addition, the degree of unsaturation of a FA on the sn-1, sn-2, or sn-3 positions may also affect the susceptibility of a lipid to peroxidation. A triglyceride with an unsaturated FA located on the sn-2 position, and SFA located on the sn-1 and sn-3 positions, would have a lower ability to be peroxidized compared to having a triglyceride with PUFA located on the sn-1 and sn-3 positions, and a SFA on the sn-2 position [110–113]. However, this may be dependent upon the method of randomization [114].

**Fig. 6:**
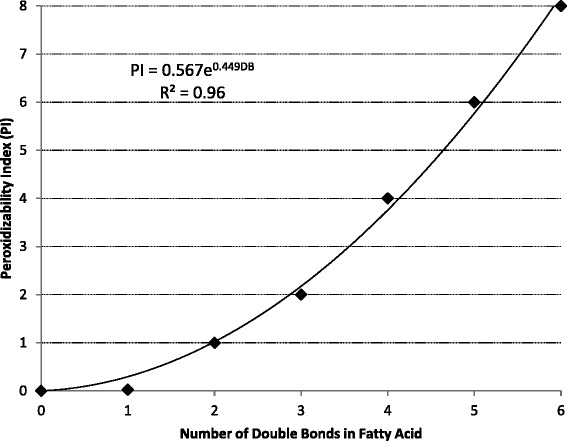
Relative susceptibility of double bonds to peroxidation [108]

Based upon an empirical measurement of oxygen consumption, and using “1” as the relative rate of oxygen consumption for linoleic acid (18:2n-6), the susceptibility of different acyl chains to peroxidative attack by oxygen as determined by Holman [108] is shown in Fig. 6. Peroxidation susceptibility among fatty acids can be very different. For example, DHA, which contains 6 double bonds, is 8-times more prone to peroxidation than linoleic acid, which has only 2 double bonds, and 320-times more susceptible to peroxidation than oleic acid which has only 1 double bond. Combining the susceptibility to peroxidation of different FA [108] with the FA composition of a lipid, it is possible to calculate a peroxidation index (**PI**) for any particular lipid where the total PI of a lipid = 0.025 × (% monoeniocs) + 1 × (% dienoics) + 2 × (% trienoics) + 4 × (% tetraenoics) + 6 × (% pentaenoics) + 8 × (% hexaenoics)]. Thus, the total PI for a particular lipid can range from 5 or less for coconut oil and tallow (low potential for peroxidation) to greater than 200 for menhaden fish oil or algae oil (high potential for peroxidation; Table 4). Belitz et al. [113] proposed an even greater impact of unsaturation on the potential of a fatty acid to be peroxidized, with the relative peroxidation rate of 18:0, 18:1, 18:2, and 18:3 being 1, 100, 1,200, and 2,500, respectively. The accuracy of these PI estimates relative to their impact on animal performance has not been evaluated.

**Table 4:** Total peroxidizability index of various lipids

Lipid source	PI^1^
Coconut	2
Tallow	5
Palm	12
Olive	13
Lard	15
Poultry	23
Canola	40
Sunflower	41
Corn	57
Soybean	65
Flaxseed	120
Menhaden	214
Algae	258

^1^Peroxidizability Index (PI) = [(0.025 × % monoeniocs) + (1 × % dienoics) + (2 × % trienoics) + (4 × % tetraenoics) + (6 × % pentaenoics) + (8 × % hexaenoics)] [108]

**Fig. 7:**
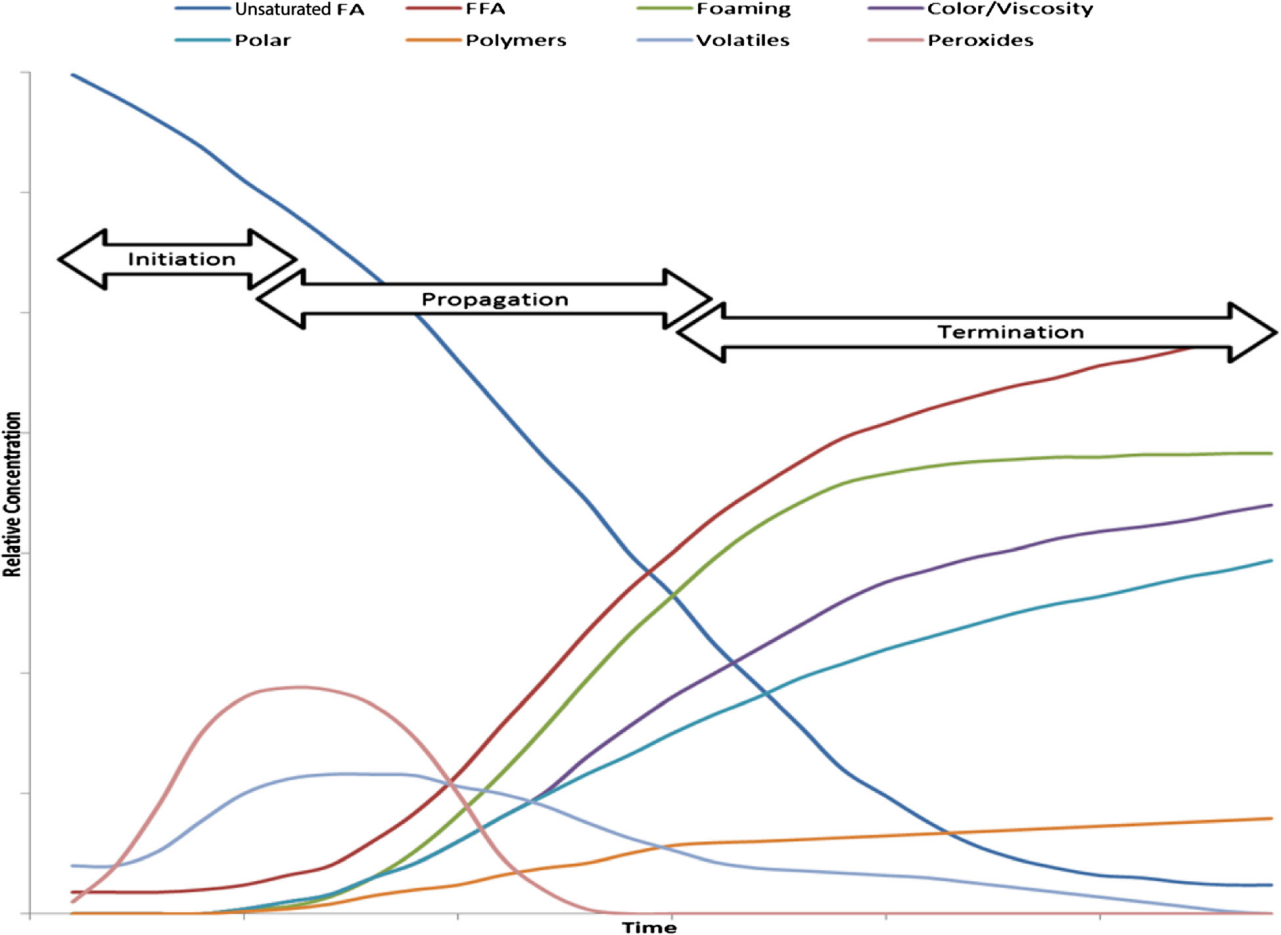
Chemical and physical changes of oil due to heating (adapted from [115])

The PI developed by Holman [108] is based solely on oxygen uptake by fatty acids and provides no specific details on which lipid peroxidation products are produced or the impact that these compounds have on energy and feeding value to pigs. Lipid hydroperoxides initially formed during the lipid peroxidation process not only have the potential to reduce its caloric value and subsequent animal health and growth performance of animals, but also result in the formation of secondary and tertiary peroxidation products (aldehydes, ketones, alcohols, hydrocarbons, volatile organic acids, and epoxy compounds) which may also negatively affect feeding value and animal productivity [18]. Consequently, the increase and subsequent decrease in the amount of various lipid peroxidation products over time during the phases of the peroxidation process increases the difficulty of accurately measuring and assessing the extent of lipid peroxidation. Because lipid peroxidation is a dynamic process, where compounds are continually produced and degraded over time, many theoretical schematics representing the production and degradation of peroxidation products have been proposed (Lubuza, 1971; [11]). Figure 7 provides a theoretical illustration of this dynamic process and further subdivides the process into the initiation, propagation, and termination phases [115].

Some of the most common chemical assays used to indicate the extent of lipid peroxidation are described in Table 5. Of these tests, peroxide value (**PV**), anisidine value (**AnV**), and thiobarbituric acid reactive substances (**TBARS**) are the most common indicative tests used in the feed industry. Peroxide value measures peroxidation products produced during the initiation phase, while AnV and TBARS are measures of peroxidation products produced during the propagation phase of peroxidation. These measures, however, do not measure compounds that remain unchanged during the peroxidation process, and hydroperoxides and aldehydes are subsequently degraded as peroxidation progresses (Fig. 7). In addition, these assays are not necessarily specific for the compounds which they were originally designed to measure [116, 117]. Consequently, new and more reliable methods utilizing HPLC or GC-MS are warranted, especially for aldehydes that are considered to be highly cytotoxic. Although malondialdehyde (**MDA**) is cytotoxic and is partially measured with the TBARS assay, the most cytotoxic and extensively studied aldehyde is 4-hydroxynonenal (**HNE;** [118, 119]). The 4-hydroxynonenal compound is an α,β-unsaturated aldehyde produced in the terminal phase of peroxidation and reacts readily with proteins, DNA, and phospholipids to affect gene expression, causes cellular and tissue damage, and has been linked to various chronic diseases [120]. Another aldehyde derived from the peroxidation of linoleic acid is 2, 4-decadienal (**DDE**), and although it is less well known and studied compared to HNE [121], it also represents a terminal lipid peroxidation compound which can be analyzed by some commercial laboratories, while HNE cannot. Polymeric compounds are also formed during the later phases of peroxidation (Fig. 7) and can be measured by size exclusion chromatography [122, 123] or by using a relative measure such as viscosity. Like many of the compounds previously described, measurement of polymers is not a common analytical procedure used for evaluating lipid quality in the animal feeds and feed ingredients, but may have important implications for assessing the safety and feeding value of lipids.

**Table 5:** Lipid peroxidation indices

Item	Description
Peroxide value (PV)	Measure of lipid peroxides and hydroperoxides.
p-Anisidine value (AnV)	Measure of the amount of the high molecular weight saturated and unsaturated aldehydes.
Thiobarbituric acid reactive substance concentration (TBARS)	Measure of carbonyl-containing secondary lipid oxidation products formed from the decomposition of hydroperoxides. Developed to detect malondialdehyde, although other carbonyl compounds can also contribute to the TBARS values.
Hexanal	Measures major secondary lipid oxidation products produced from the termination phase during the oxidation of linoleic and other ω-6 fatty acids.
2,4-decadienal (DDE)	An aldehyde derived from the peroxidation of linoleic acid.
4-hydroxynonenal (HNE)	An α, β-unsaturated lipophilic aldehyde formed from the peroxidation of polyunsaturated ω-6 fatty acids, such as linoleic or arachidonic acid.
Triacylglycerol dimers and polymers	Polymeric compounds formed during the late phases of peroxidation. Quantification of compounds based on molecular size using size exclusion chromatography or a relative value using viscosity.
Active oxygen method stability (AOM)	A predictive method where purified air is bubbled through a lipid sample at 97.8 °C, and the PV of the lipid is determined at regular intervals to determine the time required to reach a PV of 100 mEq/kg lipid (recorded as h), or the PV of the lipid is determined at a predetermined time endpoint, such as at 20 h (recorded as mEq/kg lipid).
Oil stability index (OSI)	A method whereupon air passes through a lipid under a specific temperature, at which point volatile acids decomposed from lipid peroxidation are driven out by the air and subsequently dissolved in water thereby increasing its conductivity. The conductivity of the water is constantly measured, and the OSI value is defined as the hours required for the rate of conductivity to reach a predetermined level.

Due to the high variability in composition of lipids and the phases involved in lipid peroxidation, there appears to be no single method that adequately describes or predicts lipid peroxidation [124]. Therefore, to accurately analyze the amount of lipid damage caused by peroxidation, it is necessary to determine the degree of lipid peroxidation by using more than one assay and determine peroxidation at several time intervals related to each phase of peroxidation. One such measure, TOTOX = AnV + (2 × PV) or TOTOX_TBA_ = TBARS + (2 × PV) has the advantage of combining evidence about the past history of an oil as measured by AnV with its present state as measured by PV [125]. However, despite its practical advantages, Shahidi and Wanasundra [126] indicated that TOTOX does not have a sound scientific basis because it combines variables with different dimensions. In addition, this measure fails to incorporate any compounds associated with the termination phase of peroxidation such as DDE or HNE, a measure of polymeric compounds, or a measure of remaining peroxidative potential which can be determined by active oxygen method (**AOM**) or oil stability index (**OSI**). Furthermore, no research studies have been published that have examined the potential synergistic or interactive effects between initiation, propagation, or termination phase lipid peroxidation products on the overall feeding value and quality of a lipid.

**Fig. 8:**
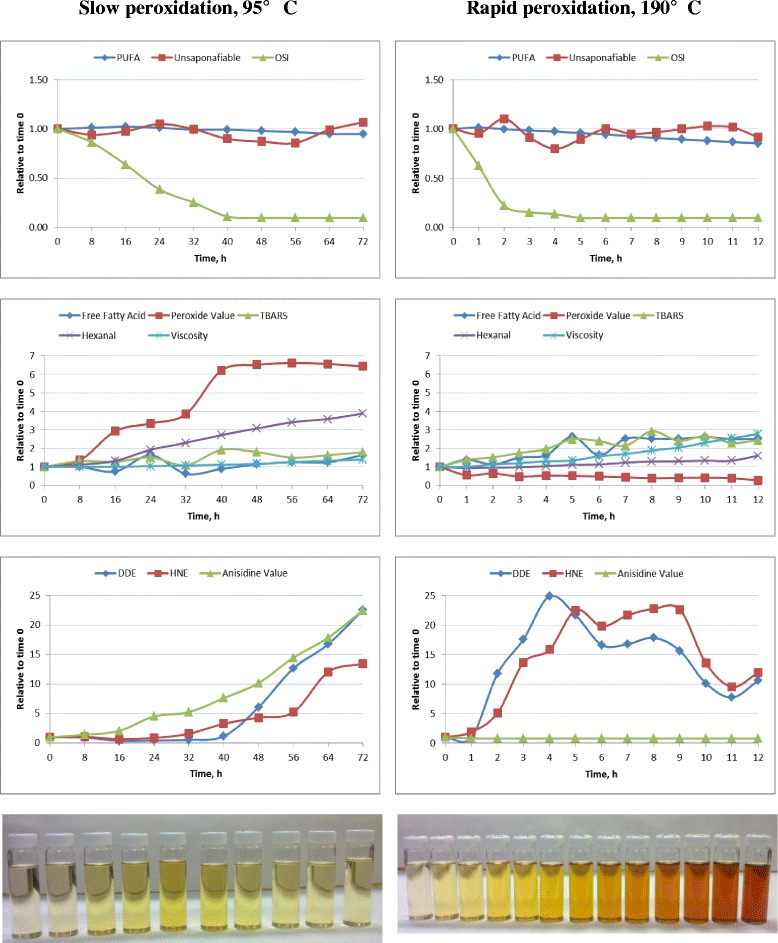
Impact of heating temperature and sampling time on indices of lipid peroxidation

Recently, Liu et al. [127] evaluated unperoxidized or peroxidized corn oil, canola oil, poultry fat, and tallow, and showed substantial changes in FFA and PUFA content depending upon the time and temperature at which the lipids were heated (95 °C for 72 h or 185 °C for 7 h). They also conducted an extensive analysis of peroxidation compounds and reported numerous correlations among various composition and peroxidation indicator and predictive measures. However, due to the potential confounding effect of lipid source composition and individual peroxidation methods, they indicated that caution should be used when interpreting their data. Because of the confounding effect of lipid source and predictive peroxidation tests, we recently conducted a time series peroxidation analysis of corn oil. For this evaluation, refined corn oil was heated at either 95 or 190 °C, using 12 L/min of air bubbled into the vesicle during the heating process, similar to that described by Liu et al. [127]. Tables 6 and 7 provide a detailed description of the composition and peroxidation measures of heated corn oil at each time point, while Fig. 8 shows the relative changes in various peroxidation measures over the course of the experiment compared to the unheated corn oil. When corn oil was heated to 95 °C, there was little impact on PUFA or unsaponifiable content (Fig. 8). There were, however, relatively large increases in PV, hexanal, AnV, DDE, and HNE, but small changes in TBARS, FFA, or viscosity, corresponding to the reduction in OSI. When corn oil was heated to 190 °C, there was little change in unsaponifiable content, but there was a steady decline in the relative amount of PUFA, and a rapid decrease in OSI. Heating corn oil to 190 °C had little impact on AnV or hexanal concentrations, but increased FFA, TBARS, and viscosity, and decreased PV compared with the original corn oil. Over time, DDE and HNE content followed a bell-shaped curve response. Although subjective, the color of the corn oil when heated at 95 °C appeared to darken and then lighten over time, while the color of the corn oil when heated at 190 °C appeared to steadily darken. These color changes are likely due to the generation and losses of volatile peroxidation compounds over time and due to concentration of polymeric compounds for the corn oil heated to 190 °C. The changes in the various lipid peroxidation measures over time clearly show that peroxidation occurred when the corn oil was heated at either temperature, but depending upon temperature, the rate of production and concentrations of peroxidation compounds was dramatically different. These data confirm the complexity of the peroxidation process and the challenges of interpreting results from various peroxidation measures as described by others.

**Table 6:** Composition of corn oil heated at 95 °C with 12 L/min air flow

Items	Sampling time, h									
Criterion	0	8	16	24	32	40	48	56	64	72
Anisidine value	0.24	0.34	0.50	1.09	1.26	1.83	2.44	3.48	4.29	5.40
Crude fat, %	>99.75	>99.75	>99.75	>99.75	>99.75	>99.75	>99.75	>99.75	>99.75	>99.75
DDE^1^, mg/mL	56.6	52.8	21.5	24.2	30.5	65.7	343.9	716.8	948.7	1276.4
Free fatty acids, %	1.12	1.12	0.83	1.83	0.70	0.98	1.27	1.41	1.40	1.84
Hexanal, μg/g	1.70	1.90	2.24	3.27	3.90	4.61	5.22	5.79	6.08	6.60
HNE^2^, μg/g	2.0	2.2	1.4	1.8	3.2	6.6	8.7	10.5	24.1	27.0
Insoluble, %	<0.15	<0.15	<0.15	<0.15	<0.15	<0.15	<0.15	<0.15	<0.15	<0.15
Moisture, %	<0.1	<0.1	<0.1	<0.1	<0.1	<0.1	<0.1	<0.1	<0.1	<0.1
Peroxide value, mEq/kg	2.11	2.87	6.17	7.06	8.12	13.10	13.75	13.94	13.85	13.57
TBARS^3^, mg MDA^4^ eq/g oil	0.018	0.023	0.023	0.027	0.020	0.034	0.032	0.027	0.029	0.032
Unsaponafiable, %	0.78	0.73	0.76	0.82	0.78	0.70	0.68	0.67	0.77	0.83
Viscosity, cP @ 20C	56.6	56.3	56.6	58.5	60.4	62.8	65.7	70.9	74.9	78.8
OSI^5^, h	10.33	8.91	6.58	3.97	2.59	1.14	<1.00	<1.00	<1.00	<1.00
Fatty acids, % of total fat^6^										
Pentadecanoic acid (C15:0)	0.00	0.00	0.00	0.00	0.09	0.13	0.14	0.16	0.18	0.15
Palmitic (16:0)	14.36	12.26	11.50	11.63	11.88	11.81	12.20	12.26	12.55	13.02
Palmitoleic (9c-16:1)	0.14	0.11	0.10	0.11	0.11	0.11	0.11	0.12	0.12	0.11
Margaric (17:0)	0.00	0.08	0.00	0.09	0.09	0.09	0.09	0.10	0.10	0.00
Stearic (18:0)	1.75	1.87	1.89	1.93	2.00	1.99	2.02	2.04	2.13	2.07
Oleic (9c-18:1)	28.93	29.79	29.97	30.16	30.51	30.56	30.79	31.04	31.49	31.60
Linoleic (18:2n6)	53.06	53.66	54.21	53.69	52.63	52.67	51.91	51.40	50.40	50.32
Linolenic (18:3n3)	0.89	0.90	0.92	0.91	0.85	0.87	0.82	0.81	0.77	0.75
Arachidic (20:0)	0.28	0.37	0.40	0.41	0.43	0.44	0.41	0.42	0.46	0.40
Gonodic (20:1n9)	0.26	0.33	0.35	0.34	0.36	0.36	0.34	0.36	0.00	0.35
Behenoic (22:0)	0.12	0.17	0.16	0.21	0.20	0.21	0.20	0.20	0.22	0.19
Lignoceric (24:0)	0.00	0.15	0.19	0.19	0.21	0.26	0.22	0.25	0.24	0.27

^1^2,4-decadienal

^2^4-hydroxynonenal

^3^Thiobarbituric acid reactive substances

^4^Malondialdehyde

^5^Oil stability index

^6^No myristoleic (9c-14:1), elaidic (9 t-18:1), vaccenic (11c-18:1), stearidonic (18:4n3), homo-α-linolenic(20:3n3), arachidonic [20:4n6], 3n-arachidonic (20:4n3), EPA (20:5n3), erucic [22:1n9], clupanodonic (22:5n3), DHA (22:6n3) or nervonic (24:1n9) fatty acids were detected

**Table 7:** Composition of corn oil heated at 190 °C with 12 L/min air flow

Items	Sampling time, h												
Criterion	0	1	2	3	4	5	6	7	8	9	10	11	12
Anisidine value	0.24	0.19	0.19	0.19	0.19	0.19	0.19	0.19	0.19	0.19	0.19	0.19	0.19
Crude fat, %	>99.75	>99.75	>99.75	>99.75	>99.75	>99.75	>99.75	>99.75	>99.75	>99.75	>99.75	>99.75	>99.75
DDE^1^, mg/mL	56.6	53.3	665.4	995.8	1410.1	1227.2	942.2	951.2	1009.4	885.9	573.4	437.8	599.2
Free fatty acids, %	1.12	1.55	1.27	1.68	1.82	2.95	1.82	2.82	2.82	2.82	2.94	2.80	2.81
Hexanal, μg/g	1.70	1.58	1.62	1.65	1.76	1.88	1.92	2.09	2.19	2.21	2.26	2.26	2.73
HNE^2^, μg/g	2.0	3.8	10.2	27.3	31.7	45.1	39.6	43.4	45.5	45.2	27.1	19.1	23.9
Insoluble, %	<0.15	<0.15	<0.15	<0.15	<0.15	<0.15	<0.15	<0.15	<0.15	<0.15	<0.15	<0.15	<0.15
Moisture, %	<0.1	<0.1	<0.1	<0.1	<0.1	<0.1	<0.1	<0.1	<0.1	<0.1	<0.1	<0.1	<0.1
Peroxide value, meq	2.11	1.15	1.35	0.99	1.11	1.07	1.00	0.91	0.79	0.84	0.87	0.80	0.55
TBARS^3^, mg MDA^4^ eq/g oil	0.018	0.024	0.027	0.031	0.035	0.044	0.043	0.038	0.052	0.043	0.047	0.041	0.043
Unsaponafiable, %	0.78	0.74	0.86	0.71	0.62	0.70	0.78	0.74	0.75	0.78	0.80	0.79	0.71
Viscosity, cP @ 20C	56.6	56.70	63.80	68.2	73.6	76	88.9	96	106.6	115.3	129.9	143.4	157.2
OSI^5^, h	10.3	6.5	2.3	1.6	1.4	<1.0	<1.0	1.0	<1.0	<1.0	<1.0	<1.0	<1.0
Fatty acids, % of total fat^6^													
Pentadecanoic acid (C15:0)	0.00	0.14	0.00	0.00	0.00	0.00	0.00	0.00	0.00	0.00	0.00	0.00	0.00
Palmitic (16:0)	14.36	11.48	11.98	12.19	12.20	12.43	12.62	12.91	13.19	13.28	13.54	13.93	13.84
Palmitoleic (9c-16:1)	0.14	0.11	0.11	0.11	0.11	0.11	0.11	0.11	0.12	0.12	0.12	0.12	0.13
Margaric (17:0)	0.00	0.09	0.00	0.00	0.00	0.00	0.00	0.00	0.10	0.10	0.00	0.00	0.11
Stearic (18:0)	1.75	1.91	1.95	1.99	2.03	2.06	2.07	2.12	2.20	2.24	2.24	2.26	2.32
Elaidic (9 t-18:1)	0.00	0.00	0.00	0.00	0.10	0.12	0.13	0.16	0.20	0.22	0.24	0.26	0.30
Oleic (9c-18:1)	28.93	29.96	30.65	31.08	31.33	31.75	32.01	32.50	32.74	32.97	33.46	33.68	33.98
Linoleic (18:2n6)	53.06	53.79	52.99	52.13	51.59	50.72	50.10	49.02	48.15	47.29	46.58	45.85	45.25
Linolenic (18:3n3)	0.89	0.89	0.82	0.79	0.77	0.73	0.70	0.65	0.62	0.60	0.56	0.53	0.52
Stearidonic (18:4n3)	0.00	0.00	0.00	0.10	0.11	0.13	0.17	0.20	0.22	0.24	0.28	0.30	0.31
Arachidic (20:0)	0.28	0.41	0.40	0.41	0.43	0.45	0.45	0.47	0.47	0.49	0.49	0.46	0.51
Gonodic (20:1n9)	0.26	0.00	0.34	0.00	0.36	0.00	0.00	0.39	0.39	0.40	0.00	0.39	0.00
Behenoic (22:0)	0.12	0.20	0.20	0.19	0.22	0.23	0.23	0.24	0.23	0.23	0.21	0.25	0.23
Lignoceric (24:0)	0.00	0.21	0.20	0.23	0.26	0.24	0.28	0.26	0.26	0.25	0.26	0.25	0.27

^1^2,4-decadienal

^2^4-hydroxynonenal

^3^Thiobarbituric acid reactive substances

^4^Malondialdehyde

^5^Oil stability index

^6^No myristoleic (9c-14:1), vaccenic (11c-18:1), homo-α-linolenic (20:3n3), arachidonic [20:4n6], 3n-arachidonic (20:4n3), EPA (20:5n3), erucic [22:1n9], clupanodonic (22:5n3), DHA (22:6n3) or nervonic (24:1n9) fatty acids were detected

## Lipid quality and nutritional value

Nutritionists and feed manufacturers use a variety of qualitative and quantitative methods to assess the quality of feed ingredients including physical, chemical, and biological tests. Physical evaluation of feed ingredients often includes color, smell, and taste characteristics that are qualitative criteria, but are used to identify characteristics that are thought to potentially lead to suboptimal animal performance when used in animal feeds. Chemical tests are quantitative and allow accurate estimation of energy and nutrient content as well as possible contaminants and toxic compounds. Biological evaluation of feed ingredients is the most definitive measure of the feeding value of an ingredient, but it is time consuming, expensive, involves controlled experimental procedures and the use of animals, and as a result, cannot be used routinely as part of a feed manufacturing quality control program.

As reported by van Kempen and McComas [128] and Shurson et al. [18], lipids used in animal feeds vary considerably in color, fatty acid profile, free fatty acid content, degree of unsaturation or saturation (iodine value, titer), saponification value, and impurities including moisture, insolubles, and unsaponifiables. The indices reported in these reports are general descriptors used to define lipid quality or ensure that the lipid products meet trading specifications, but provide limited information regarding their feeding value. Furthermore, these quality measures provide no information regarding the degree of lipid peroxidation of a lipid source. Therefore, additional measurements are required to assess lipid peroxidation.

A recent examination of 610 lipid samples obtained from a local feed manufacturer showed a wide range (0.1 to 180.8 meq O_2_/kg) in the extent of lipid peroxidation (as measured by PV) among sources [18], which is supported by a review of lipids by van Kempen and McComas [128]. Peroxidation also occurs in feed ingredients and complete feeds during storage and can be affected by feed processing conditions. Presence of oxygen, transition metals (e.g. Cu, Fe), heat, and light increase peroxidation and decrease PUFA and vitamin E content. Therefore, animals fed these peroxidized lipids can develop metabolic oxidative stress [129–131]. Peroxidation can also occur in the gastrointestinal tract, tissues, and cells resulting in damage which can negatively impact animal health and metabolism. Reactive oxygen species are produced endogenously by aerobic metabolism and the immune system, but reactive oxygen species can also be provided exogenously from the diet or produced in the gastrointestinal tract during digestion. At the cellular level, oxidative stress results in a cascade of events, beginning with damage or modification of cellular and subcellular membranes containing lipids, as well as damage to proteins, nucleic acids, and carbohydrates [132, 133]. Furthermore, some aldehydes (e.g., 4-hydroxyalkenals) present in peroxidized lipids are cytotoxic [118]. Peroxidative damage at the cellular level may increase cell rigidity and permeability, cause cell necrosis, impair cell function and integrity, contribute to structural damage of tissues, and increase demand for metabolic antioxidants [104, 133].

Exogenous (e.g. vitamin E, vitamin A, vitamin C) and endogenous (e.g. glutathione, vitamin C) antioxidants inhibit the production of reactive oxygen species. Metabolic oxidative stress occurs when pro-oxidants overwhelm the antioxidant capacity of an animal [134]. Therefore, animals with inadequate supplies of endogenous antioxidants relative to metabolic demand may develop metabolic oxidative stress. Although the number of studies are limited, feeding diets containing peroxidized lipids has been shown to result in negative effects on health and growth performance of swine and poultry [135, 136]. Diets containing peroxidized lipids cause reduced gain efficiency [137–139], growth rate [130, 140], increased metabolic oxidative status [130, 131], reduced energy digestibility [141, 142], increased mortality [129, 143], impaired immune function [144], and reduced meat quality [139, 145, 146]. Therefore, feeding diets containing peroxidized lipids can negatively affect overall animal health, growth performance, and meat quality.

Biological samples can be used to measure reactive compounds, indicators of biological damage, or antioxidants to determine metabolic oxidative status. Free radicals can be measured with electron spin resonance, but due to their short half-life, they are difficult to quantify and measurement requires specialized equipment. Unfortunately, this assay may detect relatively stable free radicals generated from antioxidants, and as a result, it is not specific to reactive oxygen species [147]. Furthermore, free radicals associated with peroxidation may be present at undetectable concentrations because of they are rapidly catabolized [147]. Some alternative assays to electronic spin resonance have been developed that are specific for hydroxy free radicals, but they are not utilized routinely [147]. Measurement of the amount of various peroxidation products in a biological sample may also provide information about metabolic oxidation status of an animal. Hydrogen peroxide [133], conjugated dienes [100], and TBARS have been measured as indicators of metabolic oxidation status, but the use of TBARS and conjugated dienes has been criticized because they lack specificity. Specific aldehydes, such as MDA and HNE, can also be measured in biological samples along with compounds indicative of peroxidative damage such as protein carbonyls, 8-hydroxy-deoxyguanosine, and isoprostanes [147]. However, the concentrations of these compounds in various tissues at which they are of concern have not been determined. However, Esterbauer et al. [118] suggested that HNE concentrations in biological samples greater than 100 μmol/L are cytotoxic, and concentrations between 1 to 20 μmol/L can cause inhibition of DNA synthesis, proteogenesis, and cellular growth, with concentrations less than 0.1 μmol/L representing basal physiological levels. Esterbauer et al. [118] also indicated that the concentration of MDA ranges from 0.2 to 0.8 μmol/L in normal human urine, but similar normal concentrations have not been determined for livestock or poultry. Liver damage resulting from feeding peroxidized diets can be measured indirectly using transaminase enzymes. Serum concentrations of hepatic transaminase enzymes have been used to assess hepatocytic damage or necrosis [148], and elevated levels of glutamate-oxalacetate transaminase and glutamate-pyruvate transaminase [149] or aspartate transaminase [150] in serum have been reported when pigs were fed diets containing inadequate concentrations of vitamin E, indicating that metabolic oxidative stress contributed to hepatocytic damage.

In addition to measurements of oxidative damage, specific endogenous antioxidants can be measured and used to assess metabolic oxidative status of an animal. Vitamin A and E can be measured in serum or liver, where relatively low concentrations may indicate metabolic oxidative stress. Negative correlations between vitamin E and TBARS concentrations in biological samples [151–153] indicate that vitamin E is catabolized during metabolic oxidative stress. Additional measures of endogenous antioxidants, such as glutathione and vitamin C, or the activity of enzymes such as glutathione peroxidase, catalase, and superoxide dismutase can be used as indicators of the ability of the animal to counteract metabolic peroxidative damage. A relatively low ratio of glutathione/glutathione reductase is a good indicator of metabolic oxidative stress because of an increased level of the oxidized form of glutathione [154].

Besides measuring specific antioxidants, other assays can be used to characterize overall metabolic antioxidative status. Measurement of the total radical-trapping antioxidant, ferric-oxide reducing antioxidant, and the trolox (a water soluble analog of vitamin E with antioxidant properties) equivalent antioxidant capacity have been used to determine the combined antioxidants activity of a sample [155]. Generally, these assays induce oxidative conditions and measure the oxidation of marker molecules added to the assay. However, the application of these assays on biological samples is often criticized because the accelerated pro-oxidant conditions of the assays do not reflect conditions *in vivo* [156]. Furthermore, because these assays are not specific to a single antioxidant, they may lack sensitivity to accurately reflect contributions from low-weight molecular antioxidants like α-tocopherol, ascorbic acid, or β-carotene [156].

Numerous assays can be used to partially assess the extent of metabolic oxidative stress in an animal, but no single measure can be used as a definitive indicator because of the complexity of the various physiological effects. Therefore, multiple measurements must be used to evaluate metabolic oxidative status, but the relative importance of specific measures relative to animal health and growth performance is not well understood. Unfortunately, there is also limited information about the use of various peroxidation measures to predict the ability of an animal to utilize a lipid source for energy.

## Antioxidants in animal nutrition

Antioxidants are chemical compounds that reduce lipid peroxidation, and are commonly added to feed ingredients and complete feeds for this purpose. However, antioxidants do not reverse peroxidation once it occurs [157]. There are many natural (e.g. carotenoids, flavonoids, phenolic acids, lignans, and citric acid) and synthetic (e.g. butylated hydroxytoluene, ethoxyquin, propyl gallate, tertiary-butylhydroquinone) compounds that have antioxidant properties, and several nutrients also directly serve as antioxidants (e.g. vitamin E, vitamin C, niacin, and riboflavin) or contribute (e.g. Se, P, Mn, Cu, Fe, Zn, and certain amino acids) to the metabolic antioxidant system [158]. In addition, several herbs (e.g. rosemary, clove, sage, oregano, thyme, mace, and allspice) and spices (e.g. wood smoke, black pepper, and mustard), as well as cocoa, tea, peanuts, soybeans, rice, oats, onions, and sweet potatoes contain significant antioxidant compounds [159]. Each antioxidant compound varies in effectiveness in the prevention of peroxidation and mode of action. However, exogenous antioxidants are generally classified as primary or secondary antioxidants based ontheir mode of action, but some antioxidants have several modes of action and act synergistically with other antioxidant compounds [158].

Primary antioxidants generally exist as mono- or polyhydroxy phenolic compounds with various ring substitutions, and quench free radicals, reactive intermediates of peroxidation, or reactive oxygen species to disrupt the chain reaction of peroxidation. As a result, antioxidant radicals are produced and stabilized by the delocalization of the unpaired electron around the phenolic ring [158]. Primary antioxidant radicals are deactivated by binding with other antioxidant free radicals to create dimers of antioxidant molecules, or they can be regenerated via reduction reactions with other antioxidants [158]. Carotenoids, flavonoids, phenolic acids, tocopherols, tocotrienols, lignans, butylated hydroxytoluene, butylated hydroxyanisole, ethoxyquin, propyl gallate, tertiary-butylhydroquinone, and other phenolic compounds act as primary antioxidants [158].

Secondary antioxidants reduce peroxidation by chelating pro-oxidant metal ions, reducing primary antioxidants, decomposing hydroperoxides, deactivating singlet oxygen, or acting as oxygen scavengers [158]. These types of antioxidants generally require the presence of other compounds to utilize their antioxidant effects, such as prolonging the effectiveness of phenolics and chelators that inhibit pro-oxidant effects of metals [160]. Carboxylic acid compounds such as phosphoric acid derivatives (e.g. phytic acid and polyphosphates), ethylenediamine-tetra-acetic acid, and citric acid also act as chelators to inhibit the pro-oxidant action of metals [158]. The oxidative stability of soybean oil declined with the addition of 0.3 ppm Fe [161] and 3 ppm Cu, Co, Mn, Fe, or Cr [162], but these effects were reduced by adding 0.01 % citric acid. Therefore, chelators such as citric acid are effective in reducing peroxidation in the presence of metals. Other secondary antioxidants work as reducing agents and oxygen scavengers. Vitamin C, carotenoids, some amino acids (e.g taurine), peptides, urates, and phenolic compounds function as reducing agents or oxygen scavengers [158]. Clements et al. [163] showed that adding 0.46 ppm β-carotene to soybean oil reduced the peroxide value and conjugated diene concentration when stored for 6 h at 20 °C.

Some antioxidants act synergistically when two or more antioxidants are combined resulting in total antioxidant activity exceeding the sum of individual activity of the antioxidants [158]. For example, the TOTOX value of palm oil increased during 1500 h exposure at 50 °C with the addition of either citric acid or tertiary butylhydroquinone, but was stabilized with the use of both compounds [157]. Other secondary antioxidants act synergistically by regeneration of primary antioxidants to extend the functionality of primary antioxidants. Cort [164] showed that ascorbic acid reduces tocopheroxyl radicals to allow regeneration of functional tocopherol.

Dietary addition of antioxidants, such as butylated hydroxyanisole, butylated hydroxytoluene, tocopherol, and ethoxyquin has been evaluated in humans, rodents, and livestock, but their impact on animal physiological and growth performance parameters has been inconsistent [165]. Dibner et al. [144, 166] reported reduced feed efficiency in broilers fed peroxidized poultry fat compared with birds fed unoxidized poultry fat, but the addition of ethoxyquin improved feed efficiency regardless of dietary lipid peroxidation level. Likewise, supplementation of additional antioxidants improved growth performance in pigs fed diets containing dried distillers grains with solubles, peroxidized corn oil, or peroxidized soybean oil [165, 167, 168]. In contrast, others have shown that supplementation of antioxidants have no effect on growth performance in animals under dietary oxidative stress conditions [169–173]. Relative to foods containing antioxidant capacity in human nutrition, a database for the Oxygen Radical Absorbance Capacity for selected foods [174] is available. In contrast, a database does not exist for animal feed ingredients which may contain antioxidant capacity from which to select for inclusion into diet formulation. To guide the selection of antioxidants, Wanasundara and Shahidi [158] recommended that the following factors be considered: 1) stability to processing conditions; 2) potency; 3) ease and accuracy of application; 4) synergistic effects with other antioxidants; 5) capacity for complete distribution with the feed; 6) minimize discoloration; and 7) ease of handling.

In addition to reducing lipid peroxidation during storage and processing, numerous antioxidants reduce peroxidation *in vivo*. Endogenous antioxidants have been classified as being non-enymatic or enzymatic depending on their function [175]. Vitamin E and Se are well known as essential nutrients with major roles in antioxidant defense, but vitamin A, vitamin C (ascorbic acid), riboflavin, niacin, P, amino acids (e.g. Met, Cys, Tau, Glu, Gly, and Trp), Mn, Cu, Fe, and Zn also have essential antioxidant functions. Non-enzymatic antioxidants such as vitamin A and vitamin E are provided in the diet and directly reduce lipid peroxidation. Vitamin E (α-tocopherol) interferes with the chain reaction of peroxidation by donating hydrogen to reactive oxygen species in the propagation step of peroxidation. The lipophilic characteristics of vitamin E allow it to be incorporated into cellular membranes where it can protect PUFA [176]. Vitamin E is a generic term which encompasses a group of 8 tocopherol and tocotrienol compounds. Packer et al. [176] suggested that tocotrienols have greater antioxidant activity than tocopherols in lipid membranes, but tocopherols have greater relative abundance in porcine plasma [177], porcine tissues [178], and murine tissues [179]. Antioxidant activity of the tocopherol isomers varies, with α > β > γ > δ, and is related to the quantity, position, and conformation of methyl groups on the aromatic ring [180]. The most common form of vitamin E added to swine diets is synthetic dl-α-tocopheryl acetate, because of enhanced stability relative to the free alcohol form [181]. The most potent metabolic form of vitamin E is α-tocopherol [182], and it has greater abundance *in vivo* relative to other forms [178]. The oxidation of vitamin E results in a relatively stable free radical that can be reduced by endogenous antioxidants such as ascorbic acid (vitamin C), glutathione, coenzyme-Q, or other molecules of oxidized vitamin E [183]. Ascorbic acid donates up to two electrons to reactive species for the regeneration of other antioxidants (e.g. vitamin E). Glutathione is an endogenously synthesized tri-peptide (composed of Glu, Gly, and Cys) and is oxidized in this process. Glutathione provides reducing equivalents during the elimination of peroxides and the regeneration of ascorbic acid, and also directly scavenges reactive oxygen species. Some forms of vitamin A also serve as antioxidants. However, the plasma concentration of vitamin A in humans [184] and pigs [130] is much lower than for vitamin E. There are many chemical forms of carotenoids which vary in their antioxidant activity. Lycopene has been shown to have the greatest antioxidant activity compared with 8 other carotenoids, including β-carotene [185]. Carotenoids are susceptible to peroxidation within the long chain of conjugated double bonds, and quench reactive oxygen species [184]. In addition, other non-enzymatic antioxidants include urate (radical scavenger), bilirubin (plasma antioxidant), flavonoids (plant antioxidants), plasma proteins (metal sequestration), and albumin (plasma antioxidant; [175]).

Enzymatic antioxidants include superoxide dismutase, catalase, glutathione peroxidase, glutathione reductase, which have direct roles in metabolic oxidation systems [183]. Superoxide dismutase catalyzes the reaction to convert superoxide (O^2−^) to peroxide in the cytosol (which is Cu and Zn dependent) or mitochondria (Mn dependent). Peroxides are eliminated in a reaction catalyzed by glutathione peroxidase (which contains Se as a structural component) along with glutathione. Catalase also works to eliminate peroxides, and Fe is a structural component of this enzyme. Other enzymes work to regenerate non-enzymatic antioxidants. Glutathione reductase (riboflavin is a structural component) and semidehydroascorbate reductase regenerate the reduced forms of glutathione and ascorbic acid, respectively, with reducing equivalents provided by nicotinamide adenine dinucleotide phosphate-oxidase (**NADPH**). Niacin and phosphorus are components of NADPH, which provides reducing equivalents to regenerate glutathione from its oxidized form. Sulfur-containing amino acids, including Met, Cys, Tau, and homocysteine play direct and indirect roles in the metabolic antioxidant system. Cystine plays an indirect role as a structural component and may be rate limiting for the synthesis of glutathione [186]. Methionine, Cys, and Tau directly scavenge reactive oxygen species [187], and there is inter-conversion among sulfur amino acids. For example, Met can be used to produce Cys in an irreversible process, with homocysteine as an intermediate, and Tau is synthesized from Cys [186].

In comparison to dietary antioxidants, many antioxidants are synthesized endogenously. Vitamin C is not a dietary essential for swine because adequate levels are generally synthesized endogenously, except in some instances of stress [11]. Ascorbic acid (vitamin C) donates up to two electrons to reactive species and assists in the regeneration of other antioxidants (e.g. vitamin E). Glutathione is an endogenously synthesized tri-peptide (Glu, Gly, and Cys) and is oxidized in this process. Glutathione provides reducing equivalents during the elimination of peroxides and the regeneration of vitamin C, and also directly scavenges reactive oxygen species. Reducing equivalents are provided by NADPH to regenerate glutathione (**GSH**) from its oxidized form glutathione disulfide (**GSSG**), and niacin and phosphorus are needed for NADPH synthesis. Sulfur-containing amino acids including Met, Cys, Tau, and homocysteine play direct and indirect roles in the antioxidant system. For example, Cys plays an indirect role as a structural component of GSH, and it may be rate limiting for endogenous synthesis of GSH [186]. Conversely, Met, Cys, and Tau directly scavenge reactive oxygen species [187].

## Conclusions

Lipids are complex but important energy contributing components of animal diets, with factors such as FA composition, FFA concentration, lipid quality indices, and degree of peroxidation having an effect on the ultimate feeding value of a lipid. While there is a substantial amount of information available on FA composition and FFA effects on digestion and energy content of various lipid sources, data relative to impact of MIU or NEM on the feeding value of lipids is limited. Information on accurate measurement of lipid peroxidation and its impact on animal health and performance are limited, but are essential for optimizing the use of various lipids in animal feeds. Universally accepted standards need to be developed for measuring quality and peroxidation status of lipid sources produced and used among the different segments of the food, agriculture, and lipid industries. Furthermore, given the complexity of the lipid peroxidation process and the potential interactions or synergisms among lipid peroxidation compounds, the use of combinations of lipid peroxidation assays that measure compounds at different stages of peroxidation is necessary to determine the dietary thresholds at which animal health and growth performance is impaired. Once this is known, the value of using supplemental dietary antioxidants on animal health and performance can be more completely determined.

## Abbreviations

AnV: *p*-anisidine value
AOM: Active oxyben method
DDE: 2,4-decadienal
DE: Digestible energy
DHA: Docosahexaenoic acid
EE: Ether extract
EPA: Eicosapentaenoic acid
FA: Fatty acid
FFA: Free fatty acids
GE: Gross energy
GSH: Glutathione
GSSG: Glutathione disulfide
HNE: 4-hydroxynonenal
MDA: Malondialdehyde
ME: Metabolizable energy
MIU: Moisture, insoluble, and unsaponifiables
MUFA: Monounsaturated fatty acids
NADPH: Nicotinamide adenine dinucleotide phosphate-oxidase
NE: Net energy
NEM: Nonelutable material
OSI: Oil stability index
PI: Peroxidizability index
PUFA: Polyunsaturated fatty acids
PV: Peroxide value
SFA: Saturated fatty acids
sn: Stereochemical number
TBARS: Thiobarbituric acid reactive substances
TOTOX: Total oxidation
